# Evaluation of the Key Structural Features of Various Butyrylcholinesterase Inhibitors Using Simple Molecular Descriptors

**DOI:** 10.3390/molecules27206894

**Published:** 2022-10-14

**Authors:** Ante Miličević, Goran Šinko

**Affiliations:** Institute for Medical Research and Occupational Health, Ksaverska cesta 2, HR-10 000 Zagreb, Croatia

**Keywords:** dementia, Alzheimer’s disease, inhibitor, butyrylcholinesterase, QSAR descriptor

## Abstract

In this study, we developed several QSAR models based on simple descriptors (such as topological and constitutional) to estimate butyrylcholinesterase (BChE) inhibition potency, p*K*_i_ (or pIC_50_), of a set of 297 (289 after exclusion of outliers) structurally different compounds. The models were similar to the best model that we obtained previously for acetylcholinesterase AChE and were based on the valence molecular connectivity indices of second and third order (^2^*χ^v^* and ^3^*χ^v^*), the number of aliphatic hydroxyl groups (nOH), AlogP Ghose–Crippen octanol–water partition coeff. (logP), and O-060–atom-centred fragments (Al-O-Ar, Ar-O-Ar, R..O..R and R-O-C=X). The best models with two and three descriptors yielded *r* = 0.787 and S.E. = 0.89, and *r* = 0.827 and S.E. = 0.81, respectively. We also correlated nine scoring functions, calculated for 20 ligands whose complexes with BChE we found in the Protein Data Bank as crystal structures to p*K*_i_ (or pIC_50_). The best correlations yielded PLP1 and PLP2 (Piecewise Linear Pairwise potential functions) with *r* = 0.619 and 0.689, respectively. Correlation with certain simple topological and constitutional descriptors yielded better results, e.g., ^3^*χ^v^* (*r* = 0.730), on the same set of compounds (*N* = 20).

## 1. Introduction

Butyrylcholinesterase (BChE, EC 3.1.1.8) is a serine hydrolase closely related to acetylcholinesterase (AChE, EC 3.1.1.7), with more than 50% identical amino acid sequences [1,2]. Both cholinesterases (ChEs) share the same catalytic mechanism of hydrolysis of choline esters, but their roles in the organism differ. AChE rapidly terminates nerve impulse transmission in cholinergic synapses by hydrolysing acetylcholine (ACh), while BChE lacks a clearly defined physiological function and does not have a single physiological substrate [3]. It can interact with various different endogenous substrates and xenobiotics, including the neurotransmitter ACh, serving as a backup in ACh hydrolysis when AChE is inhibited [4,5,6]. The catalytic efficiency of ChEs and substrate specificity is directly related to the amino acid composition of the active site, especially at the peripheral anionic site and the acyl pocket, e.g., AChE cannot hydrolase butyrylthiocholine, while BChE can [7]. Since BChE is not exclusive toward substrates and inhibitors, i.e., different ligand groups can be bounded at the same position in the active site of BChE, e.g., as in the case of piperidin phenylcarbamate (PDB ID: 6SAM [8]) and tryptophan-based azepan (PDB ID: 6XTA [9]), a general rule for very potent inhibitor design cannot be applied. In addition, BChE hydrolyses the ‘hunger hormone’ ghrelin, affecting the regulation of body weight gain or fat metabolism [10].

Dementia is now the seventh leading cause of mortality globally and one of those with the highest cost to society [11]. There are 55 million people living with dementia with a real risk of underdiagnoses in the population, and Alzheimer’s disease is the most common cause of dementia in people over the age of 65 [11]. With the progression of AD, the activity of AChE decreases, while BChE activity increases in a compensational manner [12]. The current palliative treatment of patients with AD includes ChE inhibitors, e.g., donepezil, rivastigmine and galantamine, as a cure has not yet been found. BChE inhibitors may also have a role in the treatment of AD in the future, mainly due to the introduction of the multi-target-directed ligand (MTDL) design strategies for the development of the molecules capable of simultaneously modulating multiple pathways in the neurodegenerative cascade [13,14]. For multi-target drug design in combination with BChE, human monoamine oxidase B (MAO-B) was also selected [14].

In our previous study, key features of AChE inhibitors were analysed to develop a reliable, but as simple as possible, QSAR model for the calculation of p*K*_i_ (or pIC_50_) [15]. The QSAR model with three simple molecular descriptors—the valence molecular connectivity index of the zero-order ^0^*χ^v^*, the number of 10-membered rings (nR10) and the number of hydroxyl groups (nOH)—yielded excellent statistics, *r* = 0.882 and S.E. = 0.89, on a set of 165 structurally different compounds. Since the range of experimental p*K*_i_ (or pIC_50_) values was 10.2, the error of estimation was 8.7% of the p*K*_i_ (or pIC_50_) range ((S.E./range of p*K*_i_ (or pIC_50_)) 100%). Additionally, the variables we used in the three-descriptor model (^0^*χ^v^*, nR10 and nOH) corresponded to the structural features of the compounds and AChE active site and their interactions through hydrogen bonds, hydrophobic interactions and cation–π and π–π interactions.

In this follow-up study, the focus was on the development of simple and reliable models, using simple molecular descriptors linked to the molecular key features of BChE inhibitors to estimate their inhibition potency toward BChE. For this purpose, we used a negative logarithm of experimentally determined inhibition potency values p*K*_i_ or pIC_50_, which for simplicity we denoted by pIn, for 297 structurally different compounds from the literature, including 4-aminoquinolines, coumarins, flavonoids, N-hydroxyiminoacetamides, huprine derivates, oximes, quinuclidines, tacrine derivates and others [3,8,9,16,17,18,19,20,21,22,23,24,25,26,27,28,29,30,31,32,33,34,35,36,37,38,39]. The range of pIn values for BChE inhibition was 8.6 (2.4–11).

Besides simple molecular descriptors, we also correlated the score results to the experimental pIn values. The score results were calculated from available crystal structures of the BChE-inhibitor complexes for a set of 20 ligands (cf. Table S1) that we found in the Protein Data Bank (PDB). Furthermore, we analysed those crystal structures intending to identify the key interactions between amino acids in the active site of BChE and inhibitor molecules.

## 2. Materials and Methods

### 2.1. Calculation of Topological Indices

Molecular descriptors were calculated using the E-DRAGON program, developed by Tetko et al. [40], which provides more than 1600 molecular descriptors (topological, constitutional, geometrical etc.) in a single run. After exclusion of highly inter-correlated descriptors (*r* > 0.99) we obtained a set of 857 descriptors that we used further on. The connectivity matrices were constructed using the *Online SMILES Translator and Structure File Generator* [41], and SMILE formulas for all the compounds studied are given in the Supplementary Materials (Table S1).

The QSAR models developed in this study are based on the topological indices ^2^*χ^v^* and ^3^*χ^v^* (the valence molecular connectivity indices of the second and third order) [42,43,44,45], which are defined as

(1) $${}^{2}\chi^{v}={\sum_{path}\left[\delta (i)\delta (j)\delta (k)\right]}^{-0.5}$$

and

(2) $${}^{3}\chi^{v}={\sum_{path}\left[\delta (i)\delta (j)\delta (k)\delta (l)\right]}^{-0.5}$$

where *δ*(*i*) is the weight (valence value) of each vertex (atom) *i* in a vertex-weighted molecular graph. The valence value, *δ*(*i*), of vertex *i* is defined as

(3) $$\delta (i)=[Z^{v}(i)-H(i)]/[Z(i)-Z^{v}(i)-1]$$

where *Z^v^*(*i*) is the number of valence electrons belonging to the atom corresponding to vertex *i, Z*(*i*) is its atomic number and *H*(*i*) is the number of hydrogen atoms attached to it. For instance, the delta values for the primary, secondary, tertiary and quaternary carbon atoms are 1, 2, 3 and 4, respectively, while for the oxygen in the OH group this equals 5 and for the NH_2_ group 3. It should be pointed out that ^2^*χ^v^* and ^3^*χ^v^* are only two of the many members from the family of valence connectivity indices *^n^χ^v^*, which differ amongst each other by path length, i.e., the number of consecutive chemical bonds. E.g., from Equation (1), it can be seen that ^2^*χ^v^* considers vertices (atoms) *i, j* and *k*, making up a path with a length of 2 (two consecutive chemical bonds). Connectivity indices are also called branching indices and are among the most used topological indices in QSPR/QSAR, e.g., ^3^*χ^v^* was very successfully used for the estimation of the stability constants of metal chelates [46,47,48].

### 2.2. Scoring Functions

The scoring of ligands was performed using BioVia Discovery Studio 2021 (BioVia Dassault Systèmes, San Diego, CA, USA) protocol SCORE with the following scoring functions: LigScore1, LigScore2, PLP1, PLP2, Jain, PMF, PMF04 [49,50,51,52] and two CHARMm-based scoring functions CDOCKER Energy and CDOCKER Interaction Energy [53,54]. Before the scoring itself, the crystal structure of a BChE complex needs to be prepared by excluding the ligand structure from the BChE complex by creating a separate sd file with the ligand’s original atom coordinates and preparing the enzyme structure with included waters by applying a CHARMm force field [53,55]. The Discovery Studio SCORE protocol analyses the position of the ligand within an enzyme, generating a score result based on predefined criteria for individual scoring functions. No docking procedure was used and the details about the definition of the scoring function and score calculations were explained previously [56].

### 2.3. Regression Calculations

Regression calculations, including the leave-one-out procedure (LOO) of cross validation, were performed using the CROMRsel program [57]. The standard error of the cross-validation estimate was defined as

(4) $$S.E._{\mathrm{cv}}=\sqrt{\sum_{i}\frac{\Delta X_{i}^{2}}{N}}$$

where Δ*X* and *N* denote cv residuals and the number of reference points, respectively.

## 3. Results and Discussion

**Figure 1:**
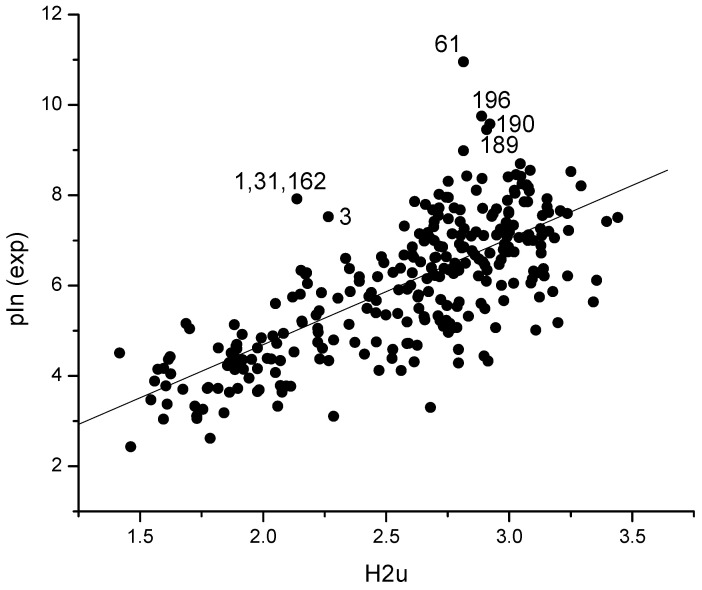
Dependence of pIn on the H2u index for 297 molecules; *r* = 0.719, S.E. = 1.05 and S.E._cv_ =1.05. Exclusion of outliers **1**, **3**, **31**, **61**, **162**, **189**, **190** and **196** from the regression yielded significantly better statistics: *r* = 0.764, S.E. = 0.93 and S.E._cv_ =0.94 (*N* = 289).

Initially, we correlated 857 molecular descriptors to experimental pIn values for 297 BChE inhibitor molecules. The single descriptor correlation with the best statistical parameters was the one that used the H2u descriptor (H autocorrelation of lag 2/unweighted), yielding *r* = 0.719, S.E. = 1.05 and S.E._cv_ = 1.05 (Figure 1). The best two descriptor models used H2v (H autocorrelation of lag 2/weighted by van der Waals volume) and nROH (number of aliphatic hydroxyl groups), and yielded *r* = 0.789, S.E. = 0.93 and S.E._cv_ = 0.94. In addition, the best three-descriptor model, H1v (H autocorrelation of lag 1/weighted by van der Waals volume), nCs (number of total secondary carbon atoms (sp^3^ hybridisation)) and nROH, resulted in *r* = 0.808, S.E. = 0.89 and S.E._cv_ = 0.92. A comparison of the standard error of the single-descriptor with the three-descriptor model resulted in ΔS.E. = 0.16 and ΔS.E._cv_ = 0.13. This means that the three-descriptor model reduced the error of prediction by about 12% in comparison to the single-descriptor model, but only 2% in comparison with the two-descriptor model.

The single-descriptor model, using H2u, indicated to the outliers present in the set (**1**, **3**, **31**, **61**, **162**, **189**, **190** and **196** in Figure 1 and Table S1), which are confirmed with the two-descriptor model, using H2v and nROH (Figure S1). After exclusion of the mentioned entries, the initial data set was reduced to 289 compounds and the statistics for one-, two- and three-descriptor models (Models 1, 2 and 3 in Table 1) improved by 10–15%. The poor fit of outliers to the model may be caused by an error in inhibition measurements or some specificities in the structure of these ligands and their interaction with the BChE active site.

**Figure 2:**
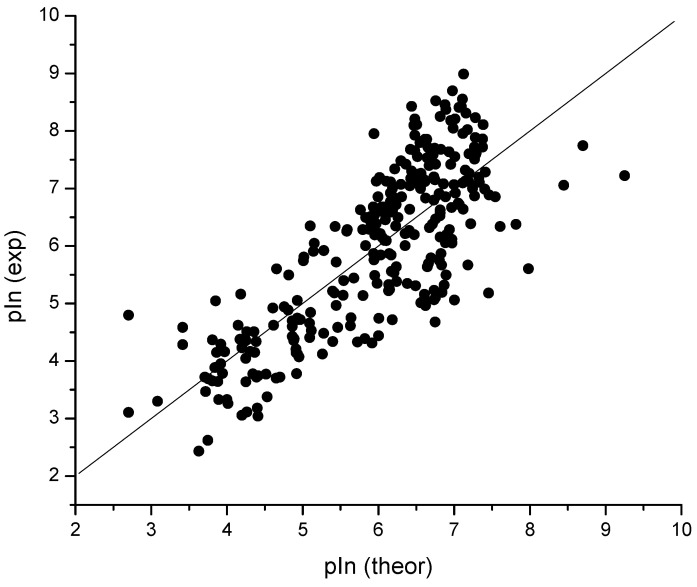
Plot of experimental vs. calculated (Model 6, Table 1) pIn values; *r* = 0.787, S.E. = 0.89 and S.E._cv_ = 0.90 (*N* = 289).

Furthermore, to simplify QSAR models, i.e., to obtain a clearer picture of the key structural features affecting the inhibition capability of the studied molecules, we excluded complex descriptors that are hard to interpret (such as 2D autocorrelations, 3D-MoRSE and WHIM descriptors). From the rest of the 336 simple descriptors (such as topological and constitutional descriptors, and molecular properties) connectivity indices ^2^*χ^v^* and ^3^*χ^v^* yielded the best single-descriptor models (S.E. = 1.02 and 1.03 for models 4 and 5, respectively, Table 1). Slightly worse statistics, S.E. = 1.07, were yielded by the model based on AlogP (Ghose–Crippen octanol–water partition coefficient (logP)). As the second descriptor to yield the best two-descriptor models either from the pool of 857 or 336 descriptors, the CROMRsel program [57] selected a number of aliphatic hydroxyl groups, nROH (Models 2, 6 and 7; Table 1; Figure 2).

These results are similar to those in our previous study [15], where the class of connectivity indices and the number of OH groups were among the most important, simple and easily interpretable variables. Moreover, although the models obtained out of the initial set of 857 descriptors (Models 1, 2 and 3; Table 1) are about 5–10% better than the models with the same number of descriptors obtained out of 336 descriptors (Models 4–9, Table 1), they are based on R2v or H2v GETAWAY descriptors that cannot be unambiguously interpreted.

The difference in S.E. between the best single-descriptor model (Model 4, Table 1) and two-descriptor model (Model 6, Table 1) was 0.13, between the best two- and three-descriptor models (Models 6 and 8, Table 1) 0.08, between the best three- and four-descriptor models (Models 8 and 10, Table 1) 0.04, and between the best four- and five-descriptor models (Models 10 and 11, Table 1) 0.03. This means that Model 6 was 13% better than Model 4, Model 8 was 9% better than Model 6, Model 10 was 5% better than Model 8 and Model 11 was 4% better than Model 10. As the statistics improvement significantly decreases with adding the fourth and fifth descriptor (Models 10 and 11, Table 1), we were satisfied with the models with two and three descriptors. Moreover, an excessive number of descriptors with the goal of obtaining better statistics may blur the relationship we are searching for, and, consequently, the detection of the important structural characteristics of a ligand responsible for its inhibition potency.

**Figure 3:**
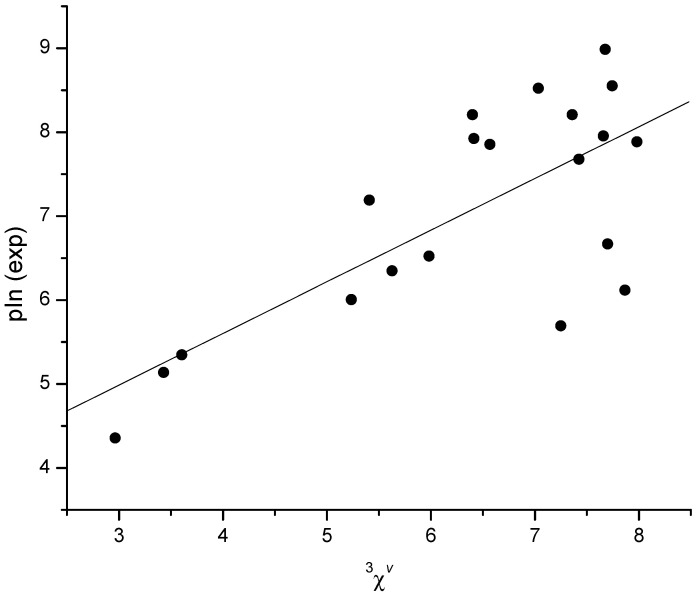
Dependence of pIn on the ^3^*χ^v^* index for 20 molecules from the Protein Data Bank. Compounds and their PDB IDs are listed in Table S1.

In our previous study [15], a series of scoring functions (LigScore1, LigScore2, PLP1, PLP2, Jain, PMF and PMF04) were correlated to pIn of AChE inhibitor ligands for which crystal structures of AChE complexes were deposited in the Protein Data Bank (PDB) [57]. Here, out of the 289 studied ligands, we found crystal structures for only 20 ligand complexes with BChE in the PDB as pdb files (*N* = 20, Table S1). The scores derived by scoring functions, with the addition of two more functions—CDOCKER Energy and CDOCKER Interaction Energy [54]—along with calculated 336 simple descriptors were correlated to pIn of 20 molecules. The best single-descriptor correlation was the one using JGT (global topological charge index), yielding *r* = 0.782, S.E. = 0.80 and S.E._cv_ = 0.87. Even the use of the number of hydrogen atoms (nH) yielded a correlation with only slightly worse statistics *r* = 0.765, S.E. = 0.83 and S.E._cv_ = 0.88, and the correlation with ^3^*χ^v^* descriptor resulted in *r* = 0.730, S.E. = 0.88 and S.E._cv_ = 0.96 (Figure 3). On the other side, among the scoring functions, PLP2 and PLP1 yielded the best correlation, resulting in *r* = 0.689 and 0.619, respectively. From Table 2, it is obvious that all of the scoring functions yielded significantly worse single-descriptor models than certain simple descriptors calculated by the E-DRAGON program [40]. Interestingly, four out of nine scoring functions, despite their complexity, yielded very poor results, i.e., *r* < 0.5.

### Interactions in BChE–Inhibitor Complexes

**Figure 4:**
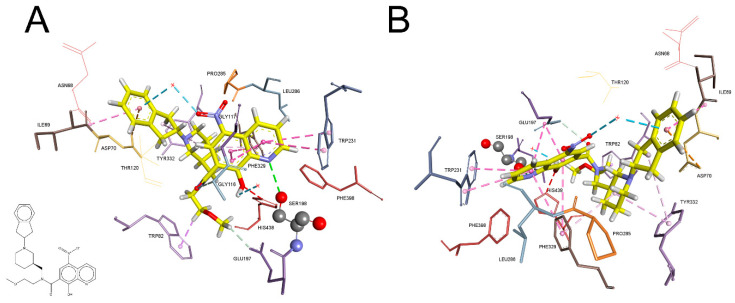
The crystal structure of the complex of a potent inhibitor with human BChE. *N*-[[(3*R*)-1-(2,3-dihydro-1*H*-inden-2-yl)piperidin-3-yl]methyl]-8-hydroxy-*N*-(2-methoxyethyl)-5-nitroquinoline-7-carboxamide (PDB ID: 4XII) [32]. Frontal view (**A**) and rotated view (**B**). Ligand carbon atoms are yellow, oxygen atoms are red and nitrogen atoms are blue. Catalytic Ser198 is represented as ball and stick. Interactions with BChE active site residues are represented as dashed lines: hydrophobic (purple or pink), hydrogen bonds (green or teal) and electrostatic (orange).

We used a crystal structure of the BChE complex with *N*-[[(3*R*)-1-(2,3-dihydro-1*H*-inden-2-yl)piperidin-3-yl]methyl]-8-hydroxy-*N*-(2-methoxyethyl)-5-nitroquinoline-7-carboxamide (Figure 4, **58** in Table S1) as an example of the relation of simple molecular descriptors in our models (Table 1) with the key interactions between amino acids in BChE active site and inhibitor molecules [32] (PDB ID: 4XII). Knez et al. reported a nanomolar ligand (*K*_i_ = 215 nM) with an 8-hydroxyquinoline group positioned in the acyl pocket stabilised by Trp231 and nearby Phe329 via π–π interactions (Figure 4). Additional stabilisation of the 8-hydroxyquinoline group comes from hydrophobic amide π stacking from Gly116 and Gly117, which strengthens the position of this group in the acyl pocket. The ligand methoxyethyl group is positioned in the choline-binding site, creating a hydrophobic interaction with the indole ring of Trp82 and hydrogen bond with Glu197. Piperidine moiety is positioned between Phe329 and Tyr332, and positively charged piperidine nitrogen is in a cation–π interaction with the vicinal Tyr332. Indene moiety is stabilised via electrostatic interaction from Asp70 and hydrophobic amide π stacking from Ile69. The ligand hydroxyl group is positioned between catalytic Ser198 and His438, forming a hydrogen bond with the water molecule.

The positive correlation of the connectivity indices ^n^*χ^v^* with inhibition potency indicates that larger and more branched molecules may have more points and areas that can interact with the active site of the enzyme. Furthermore, the larger molecule often means more carbon atoms, including aromatic ones, which increase molecule hydrophobicity (AlogP), enabling better stabilisation by hydrophobic interactions within the BChE active site, as shown in Figure 4. The number of hydroxyl groups, similarly to AChE inhibition potency, proved to be an important descriptor of complex stabilisation by hydrogen bonds. In the best three-descriptor model (Model 8, Table 1), along with ^3^*χ^v^* and AlogP, the descriptor O-060 (atom-centred fragments (Al-O-Ar, Ar-O-Ar, R..O..R, R-O-C=X)) appears, also indicating the importance of the oxygen for hydrogen bonding, Figure 4. The same model using nROH, instead of O-060, yielded slightly worse results (S.E. = 0.85 for the model using ^3^*χ^v^*, AlogP and nROH).

## 4. Conclusions

This paper is a follow-up study of the development of a simple QSAR model for a reliable evaluation of the AChE inhibitor potency of a set of 165 structurally diverse compounds [15]. In the present study, connectivity indices ^2^*χ^v^* and ^3^*χ^v^* yielded the best simple single-, two- and three-descriptor models (Table 1), showing that the class of the valence molecular connectivity indices are the most valuable descriptors for evaluation of BChE inhibitor potency, as well as for evaluation of AChE inhibitor potency [15]. Similarly, the number of aliphatic OH groups, nROH, was used as the second descriptor for the same purpose (Models 2, 6 and 7; Table 1). Similarly to the case of evaluating AChE inhibitor potency [15], where the total number of OH groups (nOH) was selected in the best model, the fact that it was selected in the best models with two, three, four and five descriptors (Models 6, 7, 9, 10 and 11, Table 1) speaks in favour of its importance. Contrary to Ref. 14, the number of 10-membered rings (nR10) was not selected in any of the best models for evaluating BChE inhibition.

From the results in Table 1, it can be calculated that Models 6 and 8 can estimate pIn values with an error of 13.5% and 12.2% of the pIn range, respectively. The range of BChE pIn values was between 2.4 and 9.0, after the exclusion of entries **1**, **3**, **31**, **61**, **162**, **189**, **190** and **196** (Table S1). Thus, BChE QSAR models with two and three simple descriptors yielded significantly worse statistics than the three-descriptor AChE model (error of 8.7% [15]), although their S.E. values were similar (S.E. was 0.89 and 0.81 for Models 6 and 8, respectively, and 0.89 for the QSAR model of AChE [15]).

However, it should also be mentioned that root mean square errors, *rms*, of negative logarithms of experimental values in the pIn range of BChE were significantly higher than the corresponding errors in the case of AChE. For example, the errors in the case of AChE were 4.8% [38], 0.80% [18] and 2.9% [36], while in the same studies the errors for BChE were 5.6% [38], 1.5% [18] and 4.3% [36].

A comparison of single-descriptor QSAR models for 20 ligands whose crystal structures of complexes with BChE we found in the Protein Data Bank (*N* = 20, Table S1) showed that all of the scoring functions yielded significantly worse results, despite their complexity, than certain simple descriptors. This result is the same as the one found by our previous study on AChE [15].

## Figures and Tables

**Table 1:** Statistical parameters of the best QSAR models using the set of 857 and 336 descriptors for 289 compounds.

Model No.	No. of Descriptors	Set of Descriptors	*r*	*r*_cv_	S.E.	S.E._cv_	Molecular Descriptor
1	1	856	0.765	0.762	0.93	0.93	R2v
2	2		0.832	0.829	0.80	0.81	H2v, nROH
3	3		0.846	0.842	0.77	0.78	H2v, nROH, BLI
4	1	336	0.703	0.698	1.02	1.03	^2^ *χ^v^*
5	1		0.698	0.692	1.03	1.04	^3^ *χ^v^*
6	2		0.787	0.782	0.89	0.90	^2^*χ^v^*, nROH
7	2		0.786	0.781	0.89	0.90	^3^*χ^v^*, nROH
8	3		0.827	0.821	0.81	0.82	^3^*χ^v^*, O-060, AlogP
9	3		0.823	0.817	0.82	0.83	ZM2V, MWC03, nROH
10	4		0.845	0.839	0.77	0.78	*χ*_T_, nROH, O-060, AlogP2
11	5		0.859	0.853	0.74	0.75	piPC07, nCs, nCbH, nROH, nArOR

R2v—R autocorrelation of lag 2/weighted by van der Waals volume; H2v—H autocorrelation of lag 2/weighted by van der Waals volume; nROH—number of aliphatic hydroxyl groups; BLI—Kier benzene-likeliness index; ^2^*χ^v^* —valence connectivity index of order 2; ^3^*χ^v^* —valence connectivity index of order 3; O-060—atom-centred fragments (Al-O-Ar, Ar-O-Ar, R..O..R and R-O-C=X); AlogP—Ghose–Crippen octanol–water partition coeff. (logP); ZM2V—second Zagreb index by valence vertex degrees; MWC03—molecular walk count of order 3; *χ*_T_—total structure connectivity index; piPC07—molecular multiple path count of order 7; nCs—number of total secondary C(sp^3^); nCbH—number of unsubstituted benzene C(sp^2^); nArOR—number of aromatic hydroxyls.

**Table 2:** Statistical parameters of the single QSAR models using scoring functions for the set of 20 molecules from the Protein Data Bank.

Scoring Functions	*r*	S.E.	S.E._cv_
CDOCKER Energy	0.123	1.27	1.39
CDOCKER Interaction Energy	0.523	1.09	1.28
LigScore1	0.533	1.08	1.21
LigScore2	0.428	1.16	1.33
PLP1	0.619	1.01	1.09
PLP2	0.689	0.93	1.01
PMF	0.193	1.25	1.42
PMF04	0.067	1.28	1.47
Jain	0.600	1.03	1.14

## Data Availability

Data are available from the corresponding author.

## Supplementary Materials

The following supporting information can be downloaded at: https://www.mdpi.com/article/10.3390/molecules27206894/s1. Table S1: List of studied compounds and QSAR descriptor values for set of 297 molecules. Figure S1: Plot of experimental vs. calculated (model with two descriptors; H2v and nROH) for set of 297 molecules.
